# Crystal structure of 2-ethyl-4-methyl-1-(2-oxido-3,4-dioxo­cyclo­but-1-en-1-yl)-1*H*-imidazol-3-ium

**DOI:** 10.1107/S2056989016009725

**Published:** 2016-06-21

**Authors:** Ufuk Korkmaz, Iclal Bulut, Ahmet Bulut

**Affiliations:** a Ondokuz Mayis University, Faculty of Arts and Sciences, Department of Physics, Atakum, Samsun, Turkey; b Ondokuz Mayis University, Faculty of Arts and Sciences, Department of Chemistry, Atakum, Samsun, Turkey

**Keywords:** crystal structure, squarene, hydrogen bonding, quantum chemical calculations, non-linear optical properties

## Abstract

In the title inner salt molecule, C_10_H_10_N_2_O_3_, the four-membered cyclobutene ring is twisted by 7.1 (2)° with respect to the five-membered imidazole ring. The crystal packing exhibits an *R*_2_^2^(9) hydrogen-bonding ring motif through N—H⋯O and C—H⋯O inter­actions. The potential non-linear optical properties were studied by a computational *ab initio* calculations performed at the DFT/B3LYP/6–31++G(d,p) level of theory.

## Abstract 2

In the crystal of the inner salt of the title compound, N—H⋯O and C—H⋯O hydrogen bonds form an 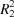
(9) ring motif.

## Chemical context   

The study of the non-linear optical (NLO) properties of organic mol­ecules and crystals are of great inter­est in physics, chemistry and applied technologies (Chemla *et al.*, 1987 ▸). Certain classes of organic compounds exhibit very pronounced NLO and electro-optical (EO) effects. Their non-linearity is based on the presence of mol­ecular units containing strongly delocalized π-electron systems with the donor and acceptor groups sited at opposite ends of the mol­ecule (Bosshard *et al.*, 1995 ▸; Kolev *et al.*, 2008 ▸). The study of the development of new non-centrosymmetric single-crystal NLO materials to obtain efficient frequency doublers is the subject of crystal engineering. In this context, some squaric acid derivatives together with cyclo­butenediones with proper substitution groups have been found to be of inter­est in terms of their high NLO responses (Kolev *et al.*, 2008 ▸).
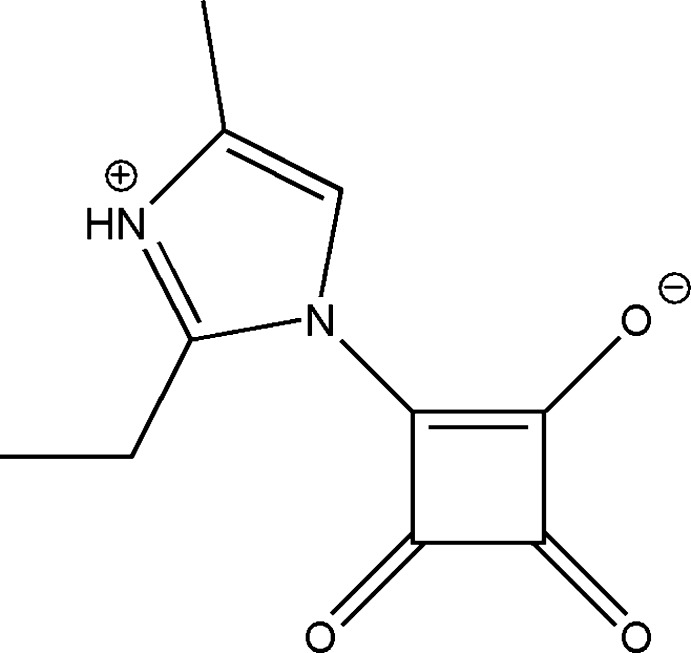


Squaric acid gives rise to two structurally different classes of derivatives, which can be described by the general mol­ecular structures 1,3-*N*-squarenes and amine-containing mol­ecule betaines (Gsänger *et al.*, 2014 ▸; Kolev *et al.*, 2005 ▸). The squarenes shows photo-chemical, photo-conductive and NLO properties and can therefore be used as electron acceptors in photo-sensitive devices (Lindsay & Singer, 1995 ▸). On the other hand, substituted betaines play an important role in NLO behavior due to their dipolar structures (Kolev *et al.*, 2004 ▸). The conversion of the N2 atom of 2-ethyl-4-methyl­imidazole into the corresponding betaine squaric acid form provides a way of enhancing the charge-transfer transition at the mol­ecular level.

This study reports a novel betain form of squaric acid with a 2-ethyl-4-methyl­imidazole mol­ecule. The crystal structure, together with its NLO properties, are reported here.

## Structural commentary   

**Figure 1:**
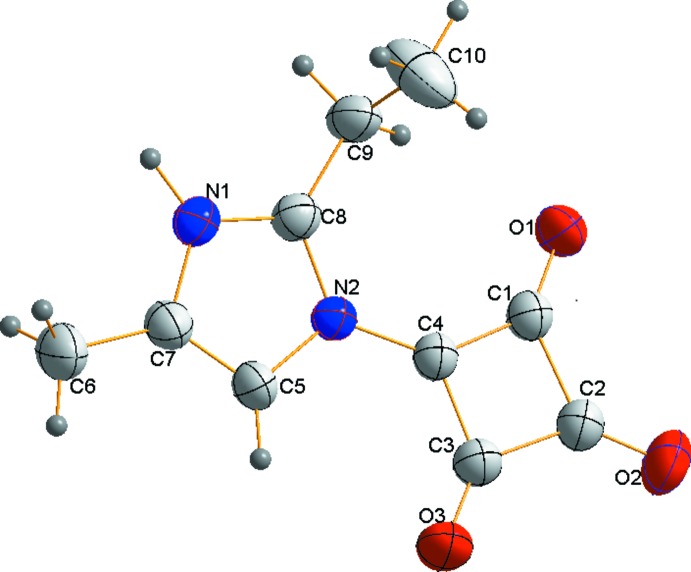
A view of the molecular structure of the title inner salt, with the atom labelling. Displacement ellipsoids drawn at the 40% probability level.

A view of the asymmetric unit is given in Fig. 1 ▸. The C1—C2—C3, C2—C1—C4 and C2—C3—C4 bond angles in the squarate ring system are almost 90°. The C1—C4—C3 bond angle is 95.0 (3)° due to the C4 atom bonding to the imidazole ring through N2 atom. The C—C distances in the planar squarate ring system of the compound reflect partial double-bond character for C1—C4 and C3—C4 [1.426 (4) and 1.440 (4) Å, respectively]. Single-bond character is observed for C1—C2 and C2—C3 [1.514 (5) and 1.516 (5) Å, respectively]. The observed bond lengths indicate a degree of delocalization in the squarate ring, as has been observed in previous studies (Kolev *et al.*, 2005 ▸; Korkmaz *et al.*, 2013 ▸). The C1—O1 and C3—O3 bond lengths are 1.234 (4) and 1.216 (4) Å, respectively. Conjugation of the squarate ring and the positively charged strong acceptor N2 result in a shortening of the carbonyl group C2=O2 bond [1.206 (4) Å]. A strong donor effect is observed for the 2-ethyl-4-methyl­imidazole group.

## Supra­molecular features   

**Figure 2:**
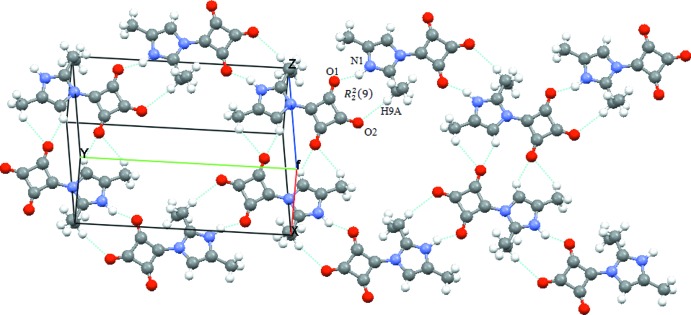
The crystal packing of the title compound, illustrating the N—H⋯O hydrogen bonds in the [010] direction together with weak C—H⋯O hydrogen bonds.

The structural properties of the mol­ecule are the result of an extensive network of hydrogen-bonding inter­actions. The N—H⋯O and C—H⋯O heteronuclear hydrogen bonds that form an 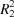
(9) ring motif contribute as both donor and acceptor to the crystal packing (Table 1 ▸, Fig. 2 ▸). The N⋯O distance should be in the region of 2.72–2.78 Å, The observed N1—H1⋯O1^i^ *D*⋯*A* distance [2.680 (3) Å; Table 1 ▸] corres­ponds to a [(+/−)CAHB] inter­action. Looking at the N⋯O distances in the symmetry-related hydrogen bonding between squarate ring systems, it can be seen that the inter­action is slightly shorter than the relevant inter­val values and is symbolized as either N^+^—H⋯ O^1\2−^ or N^+^—H⋯ O^−^ (+/−)CAHB (Korkmaz & Bulut, 2013 ▸). The C—H⋯O (Table 1 ▸, Fig. 2 ▸) inter­actions correspond to weak hydrogen bonding with an electrostatic or dispersion character according to the classification of Jeffrey (1997 ▸). In the structure, the weak C—H⋯O inter­actions are responsible for the connection between the ribbons. Therefore it can be said that the hydrogen bonds form the mol­ecular assembly, producing a uni-dimensional construction in the supra­molecular view, while the C—H⋯O inter­actions extend this to bi-dimensionality.

## Computational studies   

We have applied computational methods to evaluate the compound in terms of NLO activity. The values of the dipole moment (μ_tot_), linear polarizability (α_tot_) and first*-*order hyperpolarizability (β_tot_) of the mol­ecule were calculated at the DFT/B3LYP method level of 6-31++G(d,p) by using *Gaussian 03W* program (Frisch *et al.*, 2004 ▸). Urea is accepted as a prototype mol­ecule for non-linear optical materials and results were compared with its values (Pu, 1991 ▸). The calculation results for μ_tot_, α_tot_ and β_tot_ for urea at the same level are 3.8583 D, 4.9991 Å^3^ and 3.2637 x 10^−31^cm^5^/esu, respectively. The obtained values of μ_tot_, α_tot_ and β_tot_ for the title compound are 14.8448 D, 22.2315 Å^3^ and 6.8664 × 10^−30^ cm^5^/esu, respectively. These values are comparable with those for some of the pyridinium-betains of squaric acid (Kolev *et al.*, 2008 ▸). The value of β_tot_ appears to be much greater than that of urea. This result clearly indicates that the title compound is a strong candidate to develop a non-linear optical material. This is a prerequisite for the design of efficient second- and third-order non-linear optical materials. It should be noted that the title compound crystallized in a centrosymmetric space group (*P*2_1_/*n*).

## Synthesis and crystallization   

The title compound was synthesized according to the procedure of Schmidt *et al.* (1984 ▸). Squaric acid (H_2_Sq; 1g, 8.7 mmol) and 2-ethyl-4-methyl­imidazole (0.96 g; 8.7 mmol) were dissolved in acetic anhydride (30 cm^3^) in the molar ratio 1:1 and the solution was heated to 323 K using a controlled bath and stirred for 1 h. The reaction mixture was then cooled slowly to room temperature. The crystals formed were filtered, washed with water and methanol, and dried in air. A few days later, well-formed crystals were selected for X-ray studies. Elemental analysis for the compound (green, yield 48%) C_10_H_10_N_2_O_3_: calculated: C, 58.00; H, 5.11; N, 13.56%. Found: C, 58.25; H, 4.89; N, 13.59%. M.p. 544 K.

## Refinement   

Crystal data, data collection and structure refinement details are summarized in Table 2 ▸. The H atoms attached to C5 and N1 (H5 and H1, respectively) were located in Fourier difference maps and freely refined. The remaining H atoms were positioned geometrically (C—H = 0.96–0.97 Å) and refined using a riding model with *U*_iso_(H) = 1.2 or 1.5*U*_eq_(C).

## Supplementary Material

Crystal structure: contains datablock(s) I, global. DOI: 10.1107/S2056989016009725/xu5888sup1.cif

Structure factors: contains datablock(s) I. DOI: 10.1107/S2056989016009725/xu5888Isup2.hkl

Supporting information file. DOI: 10.1107/S2056989016009725/xu5888Isup3.cml

CCDC reference: 945212

Additional supporting information: crystallographic information; 3D view; checkCIF report

## Figures and Tables

**Table 1:** Hydrogen-bond geometry (Å, °)

*D*—H⋯*A*	*D*—H	H⋯*A*	*D*⋯*A*	*D*—H⋯*A*
N1—H1⋯O1^i^	0.97 (4)	1.73 (4)	2.680 (3)	164 (3)
C6—H6*A*⋯O1^i^	0.96	2.59	3.378 (4)	139
C9—H9*A*⋯O1	0.97	2.35	3.112 (4)	135
C9—H9*B*⋯O2^i^	0.97	2.51	3.429 (5)	158

Symmetry code: (i) 
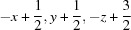
.

**Table 2:** Experimental details

Crystal data	
Chemical formula	C_10_H_10_N_2_O_3_
*M*_r_	206.20
Crystal system, space group	Monoclinic, *P*2_1_/*n*
Temperature (K)	293
*a*, *b*, *c* (Å)	4.7940 (4), 14.4120 (9), 14.5360 (9)
β (°)	93.848 (6)
*V* (Å^3^)	1002.04 (12)
*Z*	4
Radiation type	Mo *K*α
μ (mm^−1^)	0.10
Crystal size (mm)	0.34 × 0.22 × 0.22

Data collection	
Diffractometer	Agilent SuperNova (single source at offset) Eos
Absorption correction	Multi-scan (*CrysAlis PRO*; Agilent, 2011 ▸)
*T*_min_, *T*_max_	0.708, 1.000
No. of measured, independent and observed [*I* > 2σ(*I*)] reflections	5496, 3048, 1363
*R*_int_	0.048
(sin θ/λ)_max_ (Å^−1^)	0.714

Refinement	
*R*[*F* ^2^ > 2σ(*F* ^2^)], *wR*(*F* ^2^), *S*	0.080, 0.247, 1.04
No. of reflections	3048
No. of parameters	144
H-atom treatment	H atoms treated by a mixture of independent and constrained refinement
Δρ_max_, Δρ_min_ (e Å^−3^)	0.33, −0.35

Computer programs: *CrysAlis PRO* (Agilent, 2011 ▸), *SHELXS97* (Sheldrick, 2008 ▸), *SHELXL2014* (Sheldrick, 2015 ▸) and *DIAMOND* (Brandenburg, 2005 ▸).

## supplementary crystallographic information

### Crystal data

**Table:**

C_10_H_10_N_2_O_3_	*D*_x_ = 1.367 Mg m^−^^3^
*M**_r_* = 206.20	Melting point: 544 K
Monoclinic, *P*2_1_/*n*	Mo *K*α radiation, λ = 0.71073 Å
*a* = 4.7940 (4) Å	Cell parameters from 943 reflections
*b* = 14.4120 (9) Å	θ = 4.0–30.4°
*c* = 14.5360 (9) Å	µ = 0.10 mm^−^^1^
β = 93.848 (6)°	*T* = 293 K
*V* = 1002.04 (12) Å^3^	Prism, green
*Z* = 4	0.34 × 0.22 × 0.22 mm
*F*(000) = 432	

### Data collection

**Table:**

Agilent SuperNova (single source at offset) Eos diffractometer	3048 independent reflections
Radiation source: SuperNova (Mo) X-ray Source	1363 reflections with *I* > 2σ(*I*)
Detector resolution: 16.0454 pixels mm^-1^	*R*_int_ = 0.048
ω scans	θ_max_ = 30.5°, θ_min_ = 4.0°
Absorption correction: multi-scan (CrysAlis PRO; Agilent, 2011)	*h* = −6→6
*T*_min_ = 0.708, *T*_max_ = 1.000	*k* = −16→20
5496 measured reflections	*l* = −20→11

### Refinement

**Table:**

Refinement on *F*^2^	0 restraints
Least-squares matrix: full	Hydrogen site location: mixed
*R*[*F*^2^ > 2σ(*F*^2^)] = 0.080	H atoms treated by a mixture of independent and constrained refinement
*wR*(*F*^2^) = 0.247	*w* = 1/[σ^2^(*F*_o_^2^) + (0.0793*P*)^2^ + 0.493*P*] where *P* = (*F*_o_^2^ + 2*F*_c_^2^)/3
*S* = 1.04	(Δ/σ)_max_ < 0.001
3048 reflections	Δρ_max_ = 0.33 e Å^−^^3^
144 parameters	Δρ_min_ = −0.35 e Å^−^^3^

### Special details

**Table:**

Geometry. All esds (except the esd in the dihedral angle between two l.s. planes) are estimated using the full covariance matrix. The cell esds are taken into account individually in the estimation of esds in distances, angles and torsion angles; correlations between esds in cell parameters are only used when they are defined by crystal symmetry. An approximate (isotropic) treatment of cell esds is used for estimating esds involving l.s. planes.

### Fractional atomic coordinates and isotropic or equivalent isotropic displacement parameters (Å^2^)

**Table:**

	*x*	*y*	*z*	*U*_iso_*/*U*_eq_	
H5	0.783 (8)	0.546 (3)	0.562 (2)	0.060 (11)*	
H1	0.312 (8)	0.693 (3)	0.724 (2)	0.069 (12)*	
O1	0.2719 (5)	0.30379 (16)	0.74162 (16)	0.0548 (7)	
N1	0.3883 (6)	0.63979 (19)	0.69274 (18)	0.0419 (6)	
N2	0.5210 (5)	0.50284 (17)	0.65685 (16)	0.0382 (6)	
O3	0.9453 (5)	0.38166 (18)	0.54799 (18)	0.0614 (7)	
C4	0.5674 (6)	0.4072 (2)	0.6522 (2)	0.0375 (7)	
C3	0.7687 (7)	0.3595 (2)	0.6001 (2)	0.0432 (8)	
O2	0.7096 (6)	0.18740 (16)	0.63047 (19)	0.0675 (8)	
C2	0.6603 (7)	0.2691 (2)	0.6374 (2)	0.0454 (8)	
C8	0.3574 (6)	0.5506 (2)	0.7126 (2)	0.0393 (7)	
C1	0.4553 (7)	0.3249 (2)	0.6897 (2)	0.0409 (7)	
C5	0.6580 (7)	0.5660 (2)	0.6024 (2)	0.0415 (7)	
C6	0.6446 (9)	0.7446 (2)	0.5893 (2)	0.0590 (10)	
H6A	0.5446	0.7912	0.6210	0.089*	
H6B	0.8420	0.7551	0.5998	0.089*	
H6C	0.5932	0.7479	0.5244	0.089*	
C9	0.1873 (8)	0.5129 (2)	0.7852 (2)	0.0512 (9)	
H9A	0.1038	0.4547	0.7641	0.061*	
H9B	0.0369	0.5559	0.7955	0.061*	
C7	0.5737 (7)	0.6514 (2)	0.6245 (2)	0.0430 (8)	
C10	0.3587 (12)	0.4966 (3)	0.8760 (3)	0.0884 (16)	
H10A	0.2398	0.4723	0.9208	0.133*	
H10B	0.5056	0.4531	0.8665	0.133*	
H10C	0.4387	0.5543	0.8979	0.133*	

### Atomic displacement parameters (Å^2^)

**Table:**

	*U*^11^	*U*^22^	*U*^33^	*U*^12^	*U*^13^	*U*^23^
O1	0.0629 (16)	0.0385 (13)	0.0661 (15)	−0.0065 (11)	0.0278 (13)	0.0052 (11)
N1	0.0482 (16)	0.0340 (14)	0.0439 (14)	0.0004 (12)	0.0053 (12)	−0.0031 (11)
N2	0.0423 (15)	0.0330 (13)	0.0404 (12)	−0.0001 (11)	0.0110 (11)	0.0004 (10)
O3	0.0610 (17)	0.0521 (15)	0.0753 (17)	0.0066 (13)	0.0351 (14)	0.0089 (13)
C4	0.0394 (16)	0.0330 (16)	0.0405 (15)	0.0005 (13)	0.0057 (13)	0.0015 (12)
C3	0.0439 (18)	0.0396 (18)	0.0474 (17)	0.0035 (14)	0.0118 (14)	0.0021 (14)
O2	0.0790 (19)	0.0345 (14)	0.0906 (19)	0.0097 (13)	0.0179 (15)	0.0010 (13)
C2	0.0479 (19)	0.0380 (18)	0.0511 (18)	0.0041 (15)	0.0089 (15)	0.0028 (14)
C8	0.0377 (16)	0.0379 (17)	0.0426 (15)	0.0001 (13)	0.0057 (13)	−0.0020 (13)
C1	0.0448 (18)	0.0337 (16)	0.0447 (16)	−0.0004 (13)	0.0074 (14)	0.0013 (13)
C5	0.0475 (19)	0.0337 (17)	0.0446 (16)	−0.0016 (14)	0.0128 (15)	0.0027 (13)
C6	0.081 (3)	0.038 (2)	0.060 (2)	−0.0057 (18)	0.018 (2)	0.0014 (16)
C9	0.055 (2)	0.0435 (19)	0.058 (2)	−0.0025 (16)	0.0230 (17)	−0.0024 (16)
C7	0.0524 (19)	0.0368 (17)	0.0403 (15)	−0.0042 (15)	0.0062 (14)	0.0006 (13)
C10	0.128 (5)	0.089 (3)	0.049 (2)	−0.035 (3)	0.012 (2)	0.010 (2)

### Geometric parameters (Å, º)

**Table:**

C1—O1	1.234 (4)	C8—C9	1.479 (4)
N1—C8	1.328 (4)	C5—C7	1.341 (4)
N1—C7	1.386 (4)	C5—H5	0.91 (4)
N1—H1	0.97 (4)	C6—C7	1.485 (5)
C8—N2	1.353 (4)	C6—H6A	0.9600
N2—C5	1.399 (4)	C6—H6B	0.9600
C4—N2	1.399 (4)	C6—H6C	0.9600
C3—O3	1.216 (4)	C9—C10	1.526 (5)
C1—C4	1.426 (4)	C9—H9A	0.9700
C3—C4	1.440 (4)	C9—H9B	0.9700
C2—C3	1.516 (5)	C10—H10A	0.9600
C2—O2	1.206 (4)	C10—H10B	0.9600
C1—C2	1.514 (5)	C10—H10C	0.9600
			
C8—N1—C7	111.0 (3)	N2—C5—H5	121 (2)
C8—N1—H1	127 (2)	C7—C6—H6A	109.5
C7—N1—H1	121 (2)	C7—C6—H6B	109.5
C8—N2—C5	108.7 (3)	H6A—C6—H6B	109.5
C8—N2—C4	129.1 (3)	C7—C6—H6C	109.5
C5—N2—C4	122.1 (3)	H6A—C6—H6C	109.5
N2—C4—C1	137.4 (3)	H6B—C6—H6C	109.5
N2—C4—C3	127.6 (3)	C8—C9—C10	112.5 (3)
C1—C4—C3	95.0 (3)	C8—C9—H9A	109.1
O3—C3—C4	136.1 (3)	C10—C9—H9A	109.1
O3—C3—C2	135.9 (3)	C8—C9—H9B	109.1
C2—C3—C4	88.0 (2)	C10—C9—H9B	109.1
O2—C2—C1	134.2 (3)	H9A—C9—H9B	107.8
O2—C2—C3	137.3 (3)	C5—C7—N1	106.2 (3)
C1—C2—C3	88.5 (2)	C5—C7—C6	131.9 (3)
N1—C8—N2	106.5 (3)	N1—C7—C6	121.9 (3)
N1—C8—C9	125.8 (3)	C9—C10—H10A	109.5
N2—C8—C9	127.6 (3)	C9—C10—H10B	109.5
O1—C1—C4	137.8 (3)	H10A—C10—H10B	109.5
O1—C1—C2	133.6 (3)	C9—C10—H10C	109.5
C2—C1—C4	88.6 (2)	H10A—C10—H10C	109.5
C7—C5—N2	107.6 (3)	H10B—C10—H10C	109.5
C7—C5—H5	132 (2)		
			
C8—N2—C4—C1	8.9 (6)	C4—N2—C8—C9	1.3 (5)
C5—N2—C4—C1	−173.9 (3)	N2—C4—C1—O1	0.0 (7)
C8—N2—C4—C3	−171.7 (3)	C3—C4—C1—O1	−179.5 (4)
C5—N2—C4—C3	5.6 (5)	N2—C4—C1—C2	179.6 (4)
N2—C4—C3—O3	−0.7 (6)	C3—C4—C1—C2	0.0 (3)
C1—C4—C3—O3	178.9 (4)	O2—C2—C1—O1	−0.6 (7)
N2—C4—C3—C2	−179.7 (3)	C3—C2—C1—O1	179.6 (4)
C1—C4—C3—C2	0.0 (3)	O2—C2—C1—C4	179.8 (4)
O3—C3—C2—O2	1.2 (7)	C3—C2—C1—C4	0.0 (2)
C4—C3—C2—O2	−179.8 (4)	C8—N2—C5—C7	−0.9 (4)
O3—C3—C2—C1	−179.0 (4)	C4—N2—C5—C7	−178.6 (3)
C4—C3—C2—C1	0.0 (2)	N1—C8—C9—C10	−93.9 (4)
C7—N1—C8—N2	−0.5 (3)	N2—C8—C9—C10	82.6 (4)
C7—N1—C8—C9	176.7 (3)	N2—C5—C7—N1	0.5 (4)
C5—N2—C8—N1	0.8 (3)	N2—C5—C7—C6	−179.3 (3)
C4—N2—C8—N1	178.3 (3)	C8—N1—C7—C5	−0.1 (4)
C5—N2—C8—C9	−176.3 (3)	C8—N1—C7—C6	179.8 (3)

### Hydrogen-bond geometry (Å, º)

**Table:**

*D*—H···*A*	*D*—H	H···*A*	*D*···*A*	*D*—H···*A*
N1—H1···O1^i^	0.97 (4)	1.73 (4)	2.680 (3)	164 (3)
C6—H6*A*···O1^i^	0.96	2.59	3.378 (4)	139
C9—H9*A*···O1	0.97	2.35	3.112 (4)	135
C9—H9*B*···O2^i^	0.97	2.51	3.429 (5)	158

Symmetry code: (i) −*x*+1/2, *y*+1/2, −*z*+3/2.
